# A Systematic Review of the Effects of High-Fat Diet Exposure on Oocyte and Follicular Quality: A Molecular Point of View

**DOI:** 10.3390/ijms23168890

**Published:** 2022-08-10

**Authors:** Francesca Gonnella, Fani Konstantinidou, Chiara Di Berardino, Giulia Capacchietti, Alessia Peserico, Valentina Russo, Barbara Barboni, Liborio Stuppia, Valentina Gatta

**Affiliations:** 1 Department of Psychological Health and Territorial Sciences, School of Medicine and Health Sciences, “G. d’Annunzio” University of Chieti-Pescara, 66100 Chieti, Italy; 2 Unit of Molecular Genetics, Center for Advanced Studies and Technology (CAST), “G. d’Annunzio” University of Chieti-Pescara, 66100 Chieti, Italy; 3 Faculty of Bioscience, Agri-Food and Environmental Technologies, University of Teramo, 64100 Teramo, Italy

**Keywords:** female infertility, high-fat diet, oogenesis, folliculogenesis, gene expression, epigenetics

## Abstract

Worldwide, infertility affects between 10 and 15% of reproductive-aged couples. Female infertility represents an increasing health issue, principally in developing countries, as the current inclinations of delaying pregnancy beyond 35 years of age significantly decrease fertility rates. Female infertility, commonly imputable to ovulation disorders, can be influenced by several factors, including congenital malformations, hormonal dysfunction, and individual lifestyle choices, such as smoking cigarettes, stress, drug use and physical activity. Moreover, diet-related elements play an important role in the regulation of ovulation. Modern types of diet that encourage a high fat intake exert a particularly negative effect on ovulation, affecting the safety of gametes and the implantation of a healthy embryo. Identifying and understanding the cellular and molecular mechanisms responsible for diet-associated infertility might help clarify the confounding multifaceted elements of infertility and uncover novel, potentially curative treatments. In this view, this systematic revision of literature will summarize the current body of knowledge of the potential effect of high-fat diet (HFD) exposure on oocyte and follicular quality and consequent female reproductive function, with particular reference to molecular mechanisms and pathways. Inflammation, oxidative stress, gene expression and epigenetics represent the main mechanisms associated with mammal folliculogenesis and oogenesis.

## 1. Introduction

Infertility is a multifactorial pathological condition affecting approximately 10–15% of couples, especially in industrialized regions [1]. Medically assisted reproduction techniques (ART) represent one of the recommended methods for infertility treatment, even if, contrary to popular belief, it does not necessarily guarantee a positive outcome. In fact, it has been established that 38–69% of couples who start ART treatments will not be able to procreate, even after different cycles of In Vitro Fertilization (IVF) [2]. This increasingly highlights the need to develop clinically established potential predictors of successful treatments. Thus, a key question would be whether this decline can exclusively be explained by economic and behavioral factors, as indicated by demographic reports, or by biological ones as well [3]. Infertility is a condition characterized by the inability to obtain a clinical pregnancy after 12 months of unprotected intercourse in females under the age of 35, or 6 months in those over 35 years of age [4].

**Figure 1:**
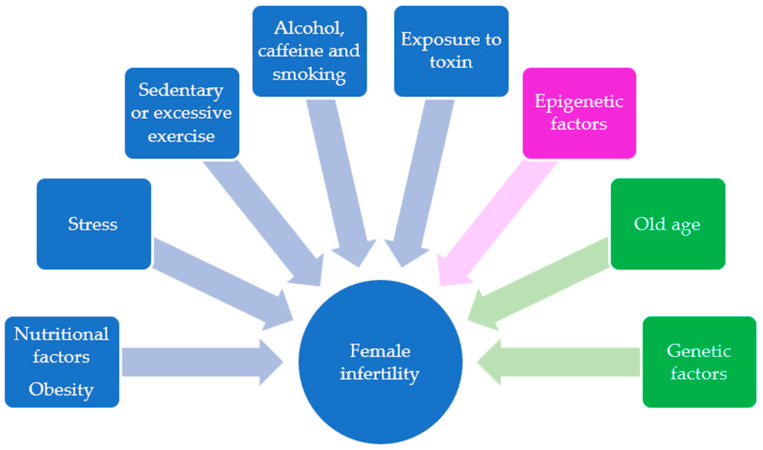
Schematic representation of lifestyle behaviors (blue). Epigenetic factors (purple) and non-modifiable risk factors (green) that contribute to female infertility.

According to the World Health Organization (WHO), about 80 million women worldwide suffer from this condition to date. The prevalence is 50% in developing countries [5]. There are many possible causes specifically linked to female infertility, such as reproductive disorders, congenital malformations, infections, hormonal dysfunction or malformations of the tubes or uterus. Impaired follicular development could also be one of the contributing factors of lower fertility levels [6,7,8]. In the mammalian ovary, follicular development is regulated by the hypothalamic–pituitary–ovary (HPO) axis, different signaling pathways and the bidirectional communication between oocytes and granulosa cells (GCs) [8,9]. Granulosa cells not only provide various energetic substrates necessary for the nourishment of the oocyte, but also have a fundamental role in the formation of corpus luteum (CL), making them indispensable for the quality of the oocyte and embryo [8]. There are many factors related to the individual’s lifestyle that are able to negatively affect female fertility, such as high consumption of caffeine and alcohol, competitive sports, stress, cigarette smoking, chronic exposure to environmental pollutants and improper eating habits [10,11,12,13]. There is accumulating evidence that non-modifiable risk factors, such as genetic mechanisms and old age, are involved in the onset of infertility [see 1 for an extensive review]. Moreover, epigenetic modifications, which are heritable alterations affected by the genetic variability and environmental influences, are associated with infertility [1,14] (Figure 1). Modern types of diet that encourage a high fat intake, such as the Atkins diet, which recommends about 50–80% of fat-derived calories; the Paleo diet, with its approximately, 28–47% of suggested dietary fat; and the Western diet, with an average of 35% of fat consumption [15], have caused an increase in obesity rates, reaching epidemic proportions [16]. An abnormal body weight, that is, a body mass index of more than 25 kg/m^2^ (according to a height/weight ^2^ ratio) [17,18]; abnormal energy intake, due to restrictions or excesses; and increased dietary consumption of carbohydrates, fatty acids, proteins, vitamins and minerals, can have a detrimental effect on ovulatory function, affecting not only the safety of gametes, but in some cases also the implantation of a healthy embryo [19]. More specifically, it has been shown that a diet containing high concentrations of saturated fatty acids (SFA) (e.g., palmitic acid), polyunsaturated fatty acids (PUFA) (e.g., linoleic acid) and monounsaturated fatty acids (MUFA) (e.g., oleic acid) could have a significant negative effect on ovarian function [20], contributing to primordial follicle loss and abnormal inflammatory responses in the ovary [21]. Furthermore, high-fat foods can also cause follicular deterioration due to increased oxidative stress in the follicular fluid (FF) [22]. Lastly, from a molecular point of view, there are several causal effects linked to obesity and low fertility. An HFD model of nutrition is associated with impaired expression and epigenetic modulation of critical genes in normal ovulatory function; metabolizing ovotoxic enzymes [23,24,25]; and ovarian inflammatory and oxidative stress markers [21]. Thus, the main objective of this systematic review of literature is to discuss the potential effects of a high-fat diet on oocyte quality and consequent female reproductive health, with particular reference to molecular mechanisms and pathways associated with mammal folliculogenesis and oogenesis.

## 2. Methods

This systematic review of the literature was carried out following the Preferred Reporting Items for Systematic Review and Meta-Analysis (PRISMA) Statement 2020 checklist guidelines. A literature search was performed using the PubMed Advanced Search Builder database. The following key phrases were used (*all variants starting with the same root are searched):

- Female infertility AND high-fat diet*;
- High fat diet AND folliculogenesis;
- High fat diet* AND-1miRNA AND ovarian follicle AND high-fat diet;
- Epigenetic OR methylation OR miRNA OR gene expression AND oocyte AND high-fat diet;
- Oxidative stress OR inflammation AND oocyte AND high-fat diet.

Only written publications in English were considered. The analyzed articles were published between 2012 and February 2022, covering a 10-year period and examining, in particular, the relationship between HFD or obesity and the oocyte, in terms of folliculogenesis and follicular growth, steroidogenesis, and oogenesis. The eligibility of the studies was mainly based on the titles and the corresponding abstracts. Except for articles regarding obesity, we excluded those that did not specifically concern the “fat-rich diet” and “oocyte” or “follicle.” The non-mammalian studies were finally discarded.

## 3. Results and Discussion

Considering the above, the initial percentage of titles in accordance with the search keywords was 72% (rounded). The complete manuscripts were then retrieved for all the selected documents, and the final inclusion was made after a thorough examination. Their reference lists were also examined and analyzed to identify other studies or potentially related information that could be further included in this review. Finally, 60 publications met the inclusion and exclusion criteria.

The data were finally grouped according to the various effects that a high-fat diet has on the molecular pathways involved in the follicles or oocytes of mammals.

### 3.1. Impact of High-Fat Diet on Gene Expression of Folliculogenesis

Consumption of fatty-acid-enriched diets and obesity have been found responsible for potentially affecting gene expression in a variety of reproductive tissues and cells, with reference to mammalian oocytes and follicles. Gene expression is regulated by two main mechanisms. The first and most well-acknowledged process is mediated through DNA, whereas the second one is epigenetic and is not directly related to DNA [26]. Most changes regarding ovarian physiology in diet-induced obesity are consequences of transcriptional changes downstream in altered leptin signaling. A recent study aimed to highlight the effect of diet-induced obesity (DIO), along with leptin administration alone on the transcriptome of murine cumulus cells (CCs) compared to controls. Mice were subjected to DIO for two time intervals, equivalent to 4 and 16 weeks, and confronted with corresponding controls exclusively exposed to a chow diet (CD). Subsequently, a model of pharmacological hyperleptinemia (LEPT) was specifically designed, by treating some of the previous CD-fed mice with high levels of leptin, and the remaining ones, which acted as controls, with saline for 16 days in order to study the transcriptome of CCs from super-ovulated mice in these conditions. In the murine model of diet-induced obesity, an overall pattern of an ultimate resistance to leptin after 16 weeks of HFD was established, as demonstrated by a combination of increased levels of gene *Socs3*—encoding the Socs3 protein, which is able to inhibit the signal transduction for various cytokines in the body, including leptin—and decreased levels of leptin receptor ObRb-pTyr985 and pJAK2, both key components of leptin signaling. Transcriptome profiling in 4-week HFD mouse CCs was like that of the LEPT CCs, especially regarding the upregulation of sets of genes actively involved in cellular trafficking and impairment in cytoskeleton organization. On the other hand, following 16 weeks of HFD, murine CCs showed changes in gene expression consistent with augmented inflammatory responses and cell morphogenesis, and decreased metabolism, mainly due to obesity-induced physiological changes. As a consequence, it was suggested that obesity conditions are able to contribute to ovarian leptin resistance and considerable time-dependent changes in gene expression in CCs, which in the early stages of obesity may be causally related to increased leptin signaling in the ovary, and in late stages are possibly results of metabolic changes taking place in the obese mothers [27]. Elevated levels of leptin were shown to stimulate higher expression of *CART*, which encodes an endogenous neuropeptide playing a key role in regulation of follicular atresia, in cattle and human granulosa cells, in overweight and obese subjects. This increase could be harmful to follicular development, having adverse effects on the GCs’ function, follicle selection, preovulatory development and ovulation.

Furthermore, in HFD-induced obese mouse models, *Cart* has been found to inhibit intracellular levels of cAMP and MAPK signaling, along with aromatase mRNA expression, leading to lower estradiol synthesis, fewer ovulated oocytes and possible subfertility [28]. It has further been reported that, following excessive consumption of fatty acids, ovarian *Edn2*, a critical gene for ovulation, and *Ece1*, called endothelin converting enzyme 1, were also dysregulated in GCs of mice during the estrous cycle. *Ece1* cleaves the Endothelin 2 (ET2) peptide to its bioactive form, and it can be the responsible for processing ET2 in the ovary. ET2 leads to contraction of the smooth muscle cells in the follicular wall of the pre-ovulatory follicle, permitting the rupture of the wall and the release of the oocyte. More specifically, a fat-rich diet tended to induce a decrease in *Edn2*, affecting the number of ovulated oocytes and overall ovulatory mechanism. On the other hand, it was evidenced that expression of *Ece1* was higher at all stages of the estrous cycle. This upregulation, combined with the dysregulation of *Edn2*, led to altered post-transcriptional processing of ET2, conducting this signal at the wrong time in the preovulatory follicles and potentially harming future ovulation [29]. In GCs of obese mice, twenty-two genes have been also reported as upregulated and twenty-six genes as downregulated in three obese models; two genetic ones called leptin knockout and leptin- receptor knockout (Lep KO and Lepr KO); and an HFD one, when compared to mice subjected to regular diet (RD). Some of these upregulated genes were present in high levels in the preovulatory follicle stage, such as *Inhbb, Stmn1* and *Hsd3b1* in obese mice. *Inhbb, Stmn1* and *Hsd3b1* are involved in metabolic homeostasis and steroidogenesis in ovarian follicles, and their increase in obese GCs induced dysregulation in GC differentiation and steroidogenesis. Other upregulated genes in obese GCs, such as *Marcks* and *Prkar2b*, were also validated as biomarkers for obese GCs. In fact, Prkar2b and Marcks proteins, actively involved correspondingly in the energy balance and adiposity regulation, and cell motility and phagocytosis, were detected by immunofluorescence staining only in the GCs of small secondary follicles from obese mice, but not in early-stage follicles from RD mice. These elevated expression levels of *Prkar2b* and *Marcks* in obese GCs of small growing follicles could indicate that they exhibit more advanced differentiation status compared to RD controls. Furthermore, in combination with the sensitivity of these genes to androgen stimulation, it was suggested that they could be considered as potential indicators of folliculogenesis abnormalities at a very early stage [30]. Obesity has been also found to have adverse effects on the expression profiles of the murine ovarian steroidogenic gene *Star*, which plays a key role in steroid hormone synthesis, and E2 receptors *Erα* and *Erβ*, which are involved in a series of physiological functions in various organ systems, such as ovarian growth and development, in granulosa cells of HFD-fed mice [31]. In particular, progressive obesity caused increases in *Star* and *Cyp11a1* mRNA expression, which catalyze the side-chain hydroxylation and cleavage of cholesterol to pregnenolone; and mRNA levels of *Erα* and *Erβ* were increased after several weeks, associated with a temporal pattern and lower levels of the E2 receptor to higher body mass, potentially resulting in decreased numbers of primordial and primary follicular [31].

These results indicate that obesity-inducing alterations of gene expression could have deleterious effects on a series of mechanisms that are essential for proper follicular functioning and maturation, contributing to the manifestation of reproductive disorders (Table 1).

#### 3.1.1. Impact of HFD on Inflammation Pathways in Folliculogenesis

A high-fat diet is known to potentially also affect inflammatory pathways present in reproductive cells. Consequently, short or long exposure to this type of diet can induce production of high levels of proinflammatory cytokines, increased infiltration of ovarian macrophages, greater penetration of immune cells and higher gene expression of inflammatory indicators in the ovaries [21,32]. This exposure is typically characterized by the presence of high levels of *TNF-α*, also known as tumor necrosis factor-α, and different types of pro-inflammatory interleukins, such as *IL-6* and *IL-8.* [33]. In particular, it has been discovered that levels of *IL-6, IL-8, TNFα* and *IL-10*, anti-inflammatory cytokines, were increased in FF of obese women, suggesting that dyslipidemia, and consequently, lipotoxicity, could be responsible for high inflammation levels from a reproductive point of view [34]. In addition, over-regulation of *FGF-12* and *PPM1L*, which play key roles in inflammation, has been reported in GCs of obese women, resulting in activation of inflammatory pathways and establishment of an unbalanced microenvironment around the oocyte in women affected by obesity [35]. Obesity has also been shown to be responsible for proinflammatory responses in the mammalian ovary. In a study conducted on obese rat models, for instance, cumulus cells of antral and preovulatory follicles showed higher expression of the *Egr-1* gene, a known proinflammatory mediator, indicating a probable obesity-related pro-inflammatory response within the ovary [33].

Based on these premises, it could be stated that obesity can also lead to significantly higher expression of known molecular inflammatory mediators in the follicle, contributing to the development of inflammation and potential irreversible damage to the follicle (Table 2).

#### 3.1.2. Impact of HFD on reactive oxygen species (ROS) Production and Oxidative Stress in Mammal Follicles

Oxidative stress is an imbalance between pro-oxidant and antioxidant factors in a cellular and tissue environment [36]. A fatty-acid-enriched diet increases the levels of the different hormones, such as FSH and LH, involved in the hypothalamic–pituitary–adrenal (HPA) axis, but also the concentration of ROS produced in the ovary and all stages of follicular growth, contributing to manifestation of oxidative stress. The relationship between oxidative stress and reproductive performance has been thoroughly studied [10,21,22], underlining that severe oxidative stress can concretely compromise follicular and ovarian development [37], and as a consequence, a balance of free radicals in the ovary is of vital importance. It has been demonstrated that due to high lipid levels, following ROS-induced lipid peroxidation in the ovaries of obese women, consequent oocyte apoptosis could be a contributing factor to significant follicular damage [38], negatively affecting ovarian function and female reproductive potential [32]. In addition, oxidative stress has been found increased by the high levels of HFD-introduced advanced glycation end products (AGEs), which end up accumulating in the plasma and FF [22]. Finally, the study of Yao J et al. indicated that oxidative stress and the expression levels of related genes were also altered in the ovaries and GCs of obese mice. More specifically, the glutathione system and *Cat* gene were activated as an antioxidant response to severe oxidative stress in the murine FF. In contrast, *Sod2*, widely known as superoxide dismutase 2, was found downregulated in obese mouse GCs compared to controls. This gene encodes the Sod2 mitochondrial protein and enzyme, which is one of the most important antioxidative defense components. Thus, its downregulation indicates a decreased capacity to eliminate ROS, mainly on behalf of the mitochondria, further contributing to oxidative stress persistence [39].

Considering the possibly causal effects between high-fat diet and obesity and increased oxidative stress levels at the follicular and ovarian levels, outcomes such as damaged follicular development and decreased reproductive potential, due to ROS-induced apoptosis, lipid peroxidation and gene expression should further be investigated to better elucidate the underlying mechanisms (Table 3).

### 3.2. Effects of HFD on Gene Expression of Oogenesis

Exposure to a high-fat diet has been associated with altered expression of genes important for normal ovulatory function, oocyte growth and apoptosis at various stages of oogenesis. For instance, modulation in expression of *Gdf9*, a growth-differentiation factor which plays a significant role in oocyte growth, has been evidenced in oocytes collected from obese murine models. More specifically, *Gdf9* was found to be more expressed in oocytes from female C57BL/6 mice fed a high-fat diet (B6-HFD) and female lethal yellow (LY) mice compared to C57BL/6 controls fed normal rodent chow (B6-ND). High levels in the ovulated oocyte could cause a *Gdf9* mRNA carryover during embryo development, leading to negative effects on development beyond the zygote stage. In addition, mRNAs encoding Raly, an RNA binding protein, were found in higher levels in oocytes from both B6-HFD and LY mice. Increase in Raly levels can be considered linked to obesity-derived alterations in RNA translation during the oocyte–zygote-transition, negatively affecting the development at the 2-cell stage and/or blastocyst [40]. It has also been reported that expression of the *Bmp15* gene, an extremely important oocyte-derived growth factor that is required for normal folliculogenesis, was upregulated in germinal vesicle (GV) and Metaphase II (MII) oocytes of HFD mice compared to mice fed subjected to a controlled diet. This could suggest that the rate of oocyte maturation may be related and susceptible to conditions of obesity, ultimately associating expression of *Bmp15* with underlying reproductive and developmental failure-related mechanisms following an HFD-induced type of obesity [41]. Other results suggest that, in GV oocytes from HFD mice, there could be an alteration in the expression of nicotinamide phosphoribosyl transferase (NAMPT) and nicotinamide adenine dinucleotide (NAD+), known regulators of NAD+ synthesis controlling numerous cell signaling pathways. More specifically, marked reductions in NAMPT’s expression and NAD+ content were noted in oocytes of high-fat-fed mice compared to those with a normal diet (ND), suggesting that NAMPT insufficiency and NAD+ reduction may possibly contribute to the overall compromised quality of oocytes subjected to HFD. In fact, the decrease in NAMPT seemed to consequently annihilate maturational progression and caused metabolic dysfunction in the oocytes [42]. Finally, a study conducted specifically on cattle cumulus oocyte complexes (COC), suggested that high levels of non-esterified fatty acids, more commonly known as NEFA, influenced Endoplasmic Reticulum (ER) stress-gene responses, along with the COC’s developmental competence and metabolism during maturation. When exposed to high levels of NEFAs, expression levels of *ATF4*, a chaperone-inducing transcription factor, and *HSPA5*, an ER-localized chaperone, were found to be significantly increased [43]. Higher expression of both of these genes was also detected in mouse COCs after they were exposed to a high-fat diet in vivo or to a higher level of lipid concentration in vitro [44]. The studies conducted on these cattle and murine models highlighted that the ER stress pathway is present in COCs exposed to high lipid content [43,44] and that the upregulation of these two ER stress-related genes could indicate that high NEFA levels can have negative impacts on protein-folding pathways, oocyte maturation, metabolic dysfunction and embryonic development [43].

Thus, existing literature sheds light on HFD-related alterations in gene expression linked to vital processes for reproductive health, such as lipid metabolism, oocyte and embryonic development, oocyte maturation and metabolic dysfunction. It could be stated, therefore, that a diet rich in fats could potentially affect not only directly the integrity and competence of the oocytes, but also the subsequent embryo metabolism and survival (Table 4).

#### Inflammation and Oxidative Stress Pathways in Oogenesis

Obesity has been also indicated to contribute to the deterioration of the ovarian environment due to accumulation of lipids, inflammation and oxidative stress. These factors can negatively affect the development and competence of oocytes, as well as later embryonic development [45]. For example, in the study conducted by Ruebel et al., significant downregulation of the *GAS7* gene was identified in Metaphase I (MI) and MII oocytes collected from overweight/obese (OW) women compared to those collected from normal weight (NW) women. *GAS7* plays a vital role in the regulation of insulin signaling, lipid metabolism, neuronal development and cell protection in embryonic stem cells, potentially altering key related developmental pathways. In MII oocytes of OW women, downregulation of *TNXIP* was further highlighted, a known oxidative stress-related gene, linked to lower oocyte quality and suggesting poorer developmental competence of OW oocytes compared to the NW ones. Higher expression of a series of genes associated with manifestation of the inflammatory state, such as *CXCL2* and *CXCL3* in GV, *IL-34* in MI and *CCL20* in MII oocytes, was also investigated in both OW and NW women. The results were confirmed by functional annotation and pathway analysis in OW oocytes compared to the NW ones, providing consistent data regarding the detected increased pro-inflammatory signaling in the ovaries and its association with related changes in gene expression in oocytes of obese mouse models [45]. These findings consistently show that obesity could be responsible for altering gene expression and contributing to the upregulation of inflammatory genes, which could lead to a chronic inflammatory response, along with decreases in oocyte quality and development. Another important aspect to consider is the effect that HFD-derived oxidative stress can have on oogenesis. It has been demonstrated, for example, that a high-fat diet can significantly increase the levels of ROS in mice oocyte. This increase has been, subsequently, associated with development of deleterious effects on oocyte maturation, fertilization and embryo development. [46]. From a molecular point of view, it has further been shown that expression of the *Sirt3* gene in both GV and MII oocytes of HFD mice is actively involved in the production of ROS by promoting the activity of *Sod2* and *Foxo3a*, well-known antioxidants, and *Idh2*, which generates NADPH necessary for scavenging ROS [46,47]. *Sirt3* gene and protein levels were found significantly lower in HFD MII oocytes compared to their lean controls, indicating that this reduction may contribute to the penetrance of oxidative stress, as well as metabolic and meiotic alterations in HFD oocytes [46]. Furthermore, it has also been reported that HFD murine GV oocytes, exposed to oxidative stress, had higher expression of pro-apoptotic genes *Bak* and *Bcl-2* compared to matched controls, suggesting a causal reduction in oocyte quality due to previous activation of early apoptosis pathways, ultimately associated with poor reproductive results [36]. A final fundamental aspect worth mentioning, regarding how oxidative stress and obesity are linked, is represented by the large presence of lipids in GV and MII mouse oocytes. Free fatty acids undergo lipid peroxidation within the oocyte, generating increases in ROS and toxic lipid peroxides, but also a decrease in the protective glutathione, establishing a state of lipotoxicity [48]. In MII oocytes of mice subjected to a fatty-acid-enriched diet, gene expression levels of *Tigar* were found drastically reduced compared to the controls. Considering that *Tigar* is normally involved in elimination of ROS during oocyte development, its downregulation is consistent with a further contribution of oxidative stress development in mammal female gametes in obesity conditions [49]. The observed manifestation of inflammation and oxidative stress due to excessive ROS production adversely affects the expression of inflammatory and antioxidant genes in mammal oocytes, potentially having harmful consequences on physiological oocyte functions, such as protective molecular antioxidant mechanisms, metabolic and meiotic alterations, overall ROS homeostasis and consequent oocyte development (Table 5).

## 4. HFD-Derived Epigenetic Effects

### 4.1. Epigenetics Mechanisms

Epigenetics is the study of heritable molecular modifications, which are responsible for the modulation of gene expression, leading to differences in the phenotype, without, however, causing modifications in the DNA sequence [50,51]. Interactions of genetic and environmental factors, which can cause changes in the epigenome, play a key role in the development of each individual. The epigenome is constantly changing throughout life and is significantly linked with the interaction between environmental stimulation and genetic activities [52]. Scientific evidence obtained from epigenetic-related studies in both animal and human models has managed to elucidate crucial roles of epigenetic mechanisms in several biological functions, such as metabolism, systemic health and growth and development [53,54]. Many types of epigenetic-related modifications have been established in mammals, which are associated with molecular processes such as DNA methylation, chromatin structure remodeling, modulation of small non-coding RNAs and histone modification. DNA methylation remains to this day the most widely studied mechanism, and it chemically consists in the attachment of small methyl groups to the CpG dinucleotides of DNA in order to produce 5-methylcytosine (5mC) [55]. Methylation, when present at the gene promoter level, tends to repress gene transcription. Histones are proteins essential for chromatin structure remodeling in eukaryotic cells. The amino-terminal tail domain of histones is susceptible to various post-transcriptional modifications, such as acetylation and methylation. Histone acetylation is linked to gene expression activation and an open chromatin state, whereas deacetylation is linked to gene silencing and histone methylation.

Another epigenetic mechanism involved in gene expression modulation is represented by non-coding RNAs (ncRNAs), including small (sncRNAs) and long ncRNAs (lncRNAs) according to their size. In mammals, sncRNAs include microRNAs (miRNAs) and Piwi RNAs. Their mechanisms of action are complex. They generally bind to the 3’-untranslated region (3′-UTR) of the target mRNA and repress protein production by silencing gene expression post-transcriptionally. They can serve as competitors, competing with other molecules by binding to the DNA; as recruiters/activators, by activating epigenetic modifiers; or as precursors, as lncRNA can be processed by RNase to produce shorter active RNAs [56].

#### 4.1.1. HFD Modifications to Gene Promoter Methylation

**Figure 2:**
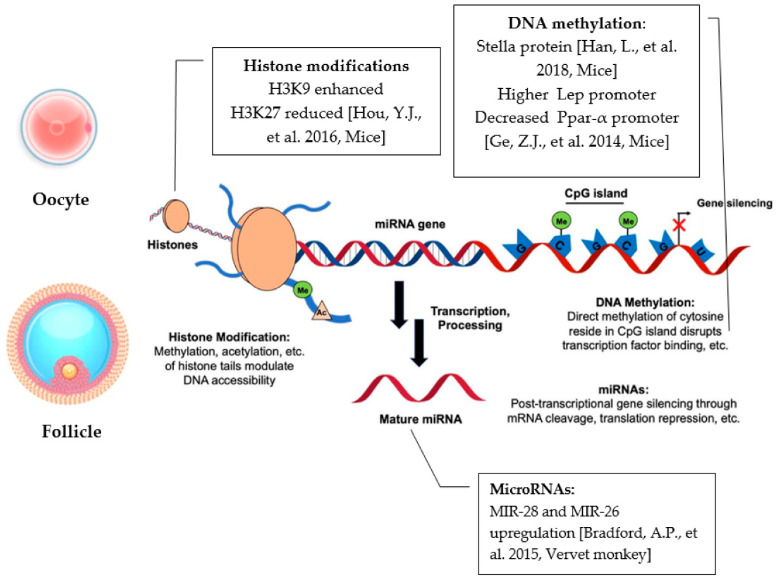
Summary figure of the main findings related to the impacts of high-fat diet on oocyte and follicular epigenetic mechanisms, DNA methylation, miRNAs and histone modifications [36,57,58,60].

Epigenetic modifications represent an important side effect that a high fat concentration diet could eventually have on the oocyte. Therefore, several scientific investigations have pointed out how an altered DNA methylation pattern could impact the oocyte quality and embryonic development. The Stella protein, for instance, has been shown to play a key role in the DNA methylation patterns of obese mice oocytes when compared to normal diet controls. Insufficiency, due to reduction in this protein’s levels, in murine MII oocytes was, in fact, suggested to be a key link between the maternal metabolic syndrome and embryonic development. Overexpression of Stella, on the other hand, caused the ability to reduce developmental defects in embryos produced from oocytes of obese mothers. Zygotes derived from obese mothers were also disrupted in normal maternal–paternal pronuclear asymmetry in CpG methylation, consistent with Stella’s proven role in inhibiting demethylation of 5mC One of its major functions, though, in terms of DNA methylation, is to maintain genome stability by silencing the expression of transposable elements (TEs). These data also demonstrated that the absence of Stella was able to generate widespread misregulation of TEs and their chimeric transcripts, due to Stella’s role as an important oocyte factor, which interposes between the effects of the mother’s diet and embryonic development and on fetal programming. In fact, the authors have shown that a decrease in Stella’s levels in the oocytes ultimately caused less damage to DNA; avoided embryonic arrest and premature demethylation at a genomic level; and overall, consequently, prevented and altered fetal growth [57]. In another study carried out in MII oocytes of HFD mice, expression of *Lep*, a metabolism-related gene, was also investigated. It is well-established that expression of this gene is regulated by DNA methylation at the promoter level. Its promoter methylation levels were found to be significantly higher in HFD oocytes compared to control diet ones, targeting gene expression and having as a result that a consequent downregulation of Lept contributed to the increase in body weight in subjects [58]. Attention was paid also to the expression of *Pparα*. Ppars are transcription factors of nuclear receptors and are divided into three main types (Pparα, Pparγ and Pparδ). They are activated endogenously by prostaglandins and fatty acids, which condition the metabolism of lipids and modulate the fatty acid oxidation (FAO), an important mechanism involved in meiotic maturation of oocytes [59]. *Pparα* is an essential factor for the regulation of systemic energy homeostasis and inflammation, and its expression is controlled by DNA methylation. As a consequence, following DNA methylation analysis of the CpG sites in oocytes of HFD mice, DNA methylation in the *Pparα* promoter was significantly decreased, partially contributing to the adverse effects of female obesity on reproduction [58] (Figure 2).

Alterations in the DNA methylation pattern in both HFD and obese oocytes have been well-documented. To our knowledge, no data were reported about HFD and methylation changes related to folliculogenesis in the last 10 years.

#### 4.1.2. HFD Modifications to microRNAs’ Expression

Several miRNAs have been analyzed in corpora lutea of vervet monkey models, in response to a high-fat and high-fructose diet (HFHF) and were associated with abnormal CL development. More specifically, MIR-28 and MIR-26 were found significantly up-regulated compared to corresponding controls subjected to a balanced diet. Combined with previous studies, it was consequently affirmed that MIR-28 could be considered responsible for limiting progesterone and testosterone production in human GCs, whilst also suppressing the release of estrogen. MIR-26a, on the other hand, was linked to minor proliferation and apoptosis of GCs. Upregulation of MIR-28 also induced downregulation in *PARL*, a critical regulator of mitochondrial morphology and function, resulting in mitochondrial abnormalities and insulin resistance. Among the targets of MIR-26, the most clearly downregulated genes were *MSMO1* and *VMA21. MSMO1* is a known mediator of LH stimulation of cholesterol biosynthesis, whereas downregulation of *VMA21*, an essential assembly chaperone of the vacuolar ATPase (V-ATPase), was associated with induction of autophagy, ER stress and metabolic syndrome, contributing to the development of metabolic syndromes (Figure 2).

Overall, the results obtained could point out how alterations in miRNA expression affect target mRNA levels for mRNAs which are important for apoptosis and follicle activation, impairing fertility and pregnancy outcomes in conditions of unbalanced nutrition choices [60]. The effects of maternal HFD on oocyte epigenetic modification, particularly on microRNAs, have yet to be systematically clarified.

#### 4.1.3. HFD Modifications on Histone Modifications

Over the past decade, a growing number of studies have indicated that maternal HFD is related to changes in various histone modifications, having significant influences on a plethora of process, such as aging, metabolic disease and the health of the offspring later in life. During oocyte growth, the chromatin structure is altered globally, and gene expression is silenced. The effects of female obesity on oocyte epigenetic modification, particularly on histone modifications, have yet to be systematically clarified. It has been observed in oocytes from mice fed either a control diet or a high-fat diet for 12 weeks, and gene mutation induced obesity models (ob/ob) mice, that lysine 9 on histone H3 methylation (H3K9-me2) was enhanced, whereas the histone modification lysine 23 on histone H3 methylation (H3K27-me2) was reduced. H3K9 and H3K27 play important roles in regulating gene expression in mitotic cells. H3K9 methylation is also an epigenetic marker of parental genome origin during early preimplantation embryo development. We conclude that epigenetic modifications were altered in both HFD and ob/ob oocytes, which may have been another reason for the low quality of oocytes from mice with obesity [36] (Figure 2).

## 5. Conclusions

In conclusion, growing evidence demonstrates that exposure to HFD has adverse effects on folliculogenesis and oogenesis. In particular, a diet rich in fats can increase inflammation and oxidative stress in the follicle and in the oocyte, causing higher expression of known molecular inflammatory mediators, ROS-induced apoptosis, lipid peroxidation and altered expression of genes involved in these pathways. Furthermore, the expression of genes related to follicular maturation, oocyte growth, metabolic homeostasis and other mechanisms, is influenced by this diet, resulting in deleterious effects on their functionality. On the other hand, HFD affects epigenetic mechanisms. As a result of aberrant methylation levels, expression levels of several genes and proteins are consequently differentially modulated, interfering with body weight and oocyte maturation, having developmental defects in embryos and resulting in low oocyte quality. In addition, miRNA upregulation in the follicle affects levels of mRNAs related to apoptosis and follicle activation. It is therefore possible to affirm, given the amount of research analyzed, that HFD impacts, first, follicular and oocyte functioning, and subsequently, female reproductive health itself.

Oxidative stress, early apoptosis and epigenetic modification might be reasons for this phenomenon. In general, the effect of the diet on the human body was traditionally observed by changes in the physiology of various organs (e.g., fatty liver, atheromatic plaques, heart failures and polycystic ovary syndrome) or at the organismal level (obesity, infertility). It is crucial to direct research studies to identifying and understanding the cellular and molecular mechanisms responsible for diet-associated diseases, including reproduction dysfunctions. This might help to clarify the confounding, multifaceted elements of infertility and uncover novel potentially curative treatments. It is also conceivable that adherence to a healthy diet positively affects female fertility. A diet based on the structure of the Mediterranean diet seems beneficial, and it could be a nutritional model able to help women with reproduction issues.

## Figures and Tables

**Table 1:** Summary table of the main findings related to the impact of a high-fat diet on gene expression of folliculogenesis.

References	Species	Main Findings
[27]	*Mice*	Upregulation of sets of genes involved in cellular trafficking and impairment in cytoskeleton organization Alterated gene expression related to inflammatory responses, cell morphogenesis and decreased metabolism
[28]	*Mice*	Higher *Cart* gene expression contributes to lower estradiol synthesis, fewer ovulated oocytes and possible subfertility
[29]	*Mice*	*Edn2* down-*, Ece1* up- gene expression and ET2 protein dysregulation influence the number of ovulated oocytes and overall ovulatory mechanism
[30]	*Mice*	*Inhbb, Stmn1,* and *Hsd3b1* higher levels modulate metabolic homeostasis and steroidogenesis in ovarian follicles; *Marcks* and *Prkar2b* are potential indicators of folliculogenesis abnormalities
[31]	*Mice*	Alteration of steroidogenic genes (*Star* and *Cyp11a1*) and E2 receptors (*Erα* and *Erβ*)

**Table 2:** A summary table of the main findings related to the impact of high-fat diet on inflammation pathways in folliculogenesis.

References	Species	Main Findings
[21]	*Mice*	Higher levels of proinflammatory cytokines, increased infiltration of ovarian macrophages
[32]	*Mice*	Greater penetration of immune cells and higher gene expression inflammatory genes
[33]	*Rat*	High levels *TNF-α, IL-6* and *IL-8* and *Egr-1* gene
[34]	*Human*	High levels *IL-6, IL-8, TNFα* and *IL-10*
[35]	*Human*	*FGF-12* and *PPM1L* over-regulation, activation of inflammatory pathways

**Table 3:** Summary table of the main findings related to the impact of high-fat diet on ROS production and oxidative stress pathways in mammal follicles.

References	Species	Main Findings
[10]	*Human*	Factors related to individual’s lifestyle—smoking
[21]	*Mice*	Abnormal inflammatory responses, inflammatory and oxidative stress markers
[22]	*Human*	Higher oxidative stress in follicular fluid
[37]	*Pig*	Oxidative stress compromises follicular and ovarian development
[38]	*Rat*	Significant follicular development damage
[32]	*Mice*	Altered ovarian function and female reproductive potential
[39]	*Mice*	Activation of glutathione system and *Cat* gene inducing antioxidant response; *Sod2* downregulation correlated to decreased capacity of eliminating ROS

**Table 4:** Summary of the main findings related to the impact of a high-fat diet on gene expression related to oogenesis.

References	Species	Main Findings
[40]	*Mice*	Higher expression of *Gdf9*, negative effects on development beyond the zygote stage Raly upregulation affects negatively the development at the 2-cell stage and/or blastocyst
[41]	*Mice*	*Bmp15* upregulation in GV and MII oocytes; developmental failure-related mechanisms
[42]	*Mice*	NAMPT reduction-induced NAD+ insufficiency; compromised quality of oocytes
[43]	*Cow*	COC’s *ATF4–HSPA5* significantly increased negative impact on protein-folding pathways, oocyte maturation, metabolic dysfunction and embryonic development
[44]	*Mice*	COC’s *Atf4–Hspa5* significantly increased

**Table 5:** Summary of the main findings related to the impacts of a high-fat diet on inflammation and oxidative stress pathways in oogenesis.

References	Species	Main Findings
[45]	*Human*	*GAS7* and *TNXIP* downregulation; Higher expression of *CXCL2, CXCL3*, *IL-34* and *CCL20*; decreased oocyte quality and development
[46]	*Mice*	*Sirt3* significatly lower may contribute to the penetrance of oxidative stress, as well as metabolic and meiotic alteration
[36]	*Mice*	Higher expression of pro-apoptotic genes *Bak* and *Bcl-2;* cause-related reduction in oocyte quality
[48]	*Mice*	Increase in ROS and toxic lipid peroxides
[49]	*Mice*	*Tigar* downregulation further contribution of oxidative stress development
